# JRC GMO-Amplicons: a collection of nucleic acid sequences related to genetically modified organisms

**DOI:** 10.1093/database/bav101

**Published:** 2015-09-30

**Authors:** Mauro Petrillo, Alexandre Angers-Loustau, Peter Henriksson, Laura Bonfini, Alex Patak, Joachim Kreysa

**Affiliations:** Molecular Biology and Genomics Unit, Joint Research Centre, European Commission, Ispra, Italy

## Abstract

The DNA target sequence is the key element in designing detection methods for genetically modified organisms (GMOs). Unfortunately this information is frequently lacking, especially for unauthorized GMOs. In addition, patent sequences are generally poorly annotated, buried in complex and extensive documentation and hard to link to the corresponding GM event. Here, we present the JRC GMO-Amplicons, a database of amplicons collected by screening public nucleotide sequence databanks by *in silico* determination of PCR amplification with reference methods for GMO analysis. The European Union Reference Laboratory for Genetically Modified Food and Feed (EU-RL GMFF) provides these methods in the GMOMETHODS database to support enforcement of EU legislation and GM food/feed control. The JRC GMO-Amplicons database is composed of more than 240 000 amplicons, which can be easily accessed and screened through a web interface. To our knowledge, this is the first attempt at pooling and collecting publicly available sequences related to GMOs in food and feed. The JRC GMO-Amplicons supports control laboratories in the design and assessment of GMO methods, providing inter-alia *in silico* prediction of primers specificity and GM targets coverage. The new tool can assist the laboratories in the analysis of complex issues, such as the detection and identification of unauthorized GMOs. Notably, the JRC GMO-Amplicons database allows the retrieval and characterization of GMO-related sequences included in patents documentation. Finally, it can help annotating poorly described GM sequences and identifying new relevant GMO-related sequences in public databases. The JRC GMO-Amplicons is freely accessible through a web-based portal that is hosted on the EU-RL GMFF website.

**Database URL**: http://gmo-crl.jrc.ec.europa.eu/jrcgmoamplicons/

## Introduction

The European Union (EU) has defined a legal framework that strictly regulates cultivation of genetically modified organisms (GMOs) and their marketing as food and feed (1–3). Implementation of EU regulations on GMOs and their traceability requirements strictly depends on the availability of reliable and reproducible analytical methods. According to Article 32 of Regulation (EC) N. 882/2004 (4) the European Union Reference Laboratory for Genetically Modified Food and Feed (EU-RL GMFF) must provide National Reference Laboratories (NRLs) with reference methods for GMO analysis. To this end the EU-RL GMFF, hosted by the Joint Research Centre (JRC) of the European Commission, has compiled a state-of-the-art catalogue of DNA-based methods and made them freely accessible in its Database of Reference Methods called GMOMETHODS (5). The GMOMETHODS database only includes methods that have been validated through a collaborative trial according to the international standard ISO 5725 (6) and/or the IUPAC protocol (Union of Pure and Applied Chemistry, 7). These methods have demonstrated performance characteristics in line with the criteria established by the European Network of GMO laboratories (ENGL) and can therefore assure reliable, reproducible, sensitive and accurate determination of GMO content in food and feed. Other applications, such as the GMO Detection Method Database (GMDD, 8), do not perform such a stringent selection and cannot therefore assure the same performance reliability for the methods that they provide.

The GMOMETHODS detection methods are currently based on the polymerase chain reaction (PCR) technology and are grouped according to the specificity of the target sequence:

- *Event-specific* methods have the highest levels of specificity, as they target regions that are unique to each DNA integration event.
- *Construct-specific* methods are specific for the transgenic constructs, as they target a region that spans artificially joined sequences, such as a coding sequence and the neighbouring control regions.
- *Element-specific* methods target DNA sequences that are solely confined to one particular sequence element of a GMO, such as the terminator of the *nopaline synthase* gene of *Agrobacterium tumefaciens* (T-nos) or a gene coding region.
- *Taxon-specific* (or *species-specific*) methods target DNA sequences that are specific to a particular taxon or species. They are used for defining the composition and relative GM content of a food/feed product at ingredient level.

For the analysis of GMOs in the food and feed chains, the GMO-control laboratories generally perform an initial ‘screening’ step. In order to reduce the list of possible GMOs present, a set of element- or construct-specific methods (also called *screening methods*) is used for detecting target sequences commonly present in known GM events. In case of positive signals, event-specific methods are employed to identify the GMOs containing the previously detected GM elements. Moreover, to assist the GMO-control laboratories in the whole screening phase, information matrices of the expected PCR amplification patterns of known GMOs for a defined range of element- and construct-specific detection methods have been developed. Some of the matrices available online, such as GMOseek (9) and GMOfinder (10), integrate information from laboratory testing and publicly available sources, such as public versions of dossiers submitted by the applicants to regulatory authorities. However, their predictions are not always reliable as elements with the same name can vary at the level of their DNA sequences in different GMOs, and a set of primers and probes designed for detecting a specific element may or may not efficiently amplify a slightly different version of the same element. Moreover, the DNA sequences themselves may not always be readily available for allowing such predictions. To avoid these shortcomings we have recently made available the JRC GMO-Matrix, a matrix-based decision support system that is unique for its reliance on DNA sequence data (11). This new interactive tool relies on data stored in the Central Core DNA Sequences Information System (CCSIS) (12), a database of annotated GM event sequences retrieved from public databases or provided by the applicants for the EU authorization procedure and therefore covering, for each event, the entire insert with their respective flanking regions. A direct comparison of the GM and methods sequences allows a more reliable *in silico* prediction of the PCR results for every combination of reference method (primers/probes) and GM event.

Given the confidential nature of some of these sequences information, the CCSIS database is not directly publicly available but processed, in the JRC GMO-Matrix, in the form of precomputed values of PCR predictions. The dependency of these simulations on the availability of complete and accurate GM sequences can limit the coverage of GM events found in the JRC GMO-Matrix, in particular for unknown or poorly characterized GMOs. For these, information can be complemented by other sources of information, e.g. by compiling experimental test results or by mining public sequence databases for transgenic targets. GMO-related sequence data may be stored in public sequence repositories as a contribution from published literature, grouped sometimes in broad sections, such as the transgenic (*tgn*) data class division of the European Nucleotide Archive (ENA) (13). Patented sequence datasets are another putative source, as GMOs are often patented. Sequence data present in patents are usually retrieved and grouped in the public databases, such as the NCBI *patnt* collection (14) or the *pat* data class division of ENA (13), but are characterized by a high level of redundancy. Few attempts have been made to address this redundancy, such as the development of the *nrnl1* database (15). Moreover, patented sequences are poorly annotated and can be linked to the corresponding GMO only by extensive review of the original documentation.

To our knowledge, there are currently no databases aimed specifically at storing GMO-related sequences. Here, we present such a resource, called JRC GMO-Amplicons. JRC GMO-Amplicons contains and makes available results of a bioinformatic pipeline that regularly screens public nucleotide sequence databanks, including patents and available whole plant genomes, through *in silico* determination of PCR amplification with the reference methods of the GMOMETHODS database.

The JRC GMO-Amplicons can be queried by control laboratories to evaluate results of their screening/identification analysis or for developing new detection methods and assessing *in silico* their primers specificity and GMO coverage. Moreover, with the advent of next-generation sequencing (NGS), the availability of an up-to-date source of sequences related to GMOs is crucial for the molecular characterization of GM events. Finally, the JRC GMO-Amplicons database can aid in annotating poorly described GM events or in recognizing new GM-related sequences in public repositories.

## Methods

### Platform deployment

The platform supporting the JRC GMO-Amplicons functions comprises three servers: a server dedicated to the database (PostgreSQL, http://www.postgresql.org/) storing all DNA sequences and related information, a server hosting a Ruby on Rails web application (http://rubyonrails.org/) that acts as a user interface, and a high-performance computer that regularly runs a compute-intensive pipeline.

### Data and resources

The GMOMETHODS database includes, as of January 2015, 140 different PCR methods, specifically 60 event-specific, 20 construct-specific, 26 element-specific and 37 taxon-specific methods.

In the current implementation, the pipeline runs on a regular basis (i.e. every 3 months) on the following public data sets:

- EBI non-redundant patent nucleotide database *nrnl1*, available as a single file at ftp://ftp.ebi.ac.uk/pub/databases/fastafiles/patent/nrnl1.gz (release of 2 January 2013)
- the NCBI *nt* collection, available as a single file at ftp://ftp.ncbi.nih.gov/blast/db/FASTA/nt.gz (release of 6 December 2014)
- the NCBI patnt collection, available as a single file at ftp://ftp.ncbi.nih.gov/blast/db/FASTA/patnt.gz (release of 6 December 2014)
- the *pat* (patent) data class division of the ENA, available as multiple em_rel_pat_*.gz files at ftp://ftp.ebi.ac.uk/pub/databases/fastafiles/emblrelease/ (release 121)
- the plant *conexp* (contig expanded) class division of the ENA, available as multiple rel_exp_con_pln_*.dat.gz files at ftp://ftp.ebi.ac.uk/pub/databases/ena/sequence/release/expanded_con (release 121)
- the *tgn* (transgenic) data class division of the ENA, available as multiple em_rel_*_tgn.gz files at ftp://ftp.ebi.ac.uk/pub/databases/fastafiles/emblrelease/ (release 121)

These data sets are regularly downloaded and installed as local copies for the analysis and updated when new release versions become available.

### Pipeline

The pipeline was written in the PHP5 (http://php.net) object-oriented programming language. Given a set of nucleic acid sequences and the array of reference methods in the GMOMETHODS database, the pipeline sequentially performs the tasks listed below:

1. *In silico* PCR simulation on the whole sequence collection with the primers pairs of the reference methods, by running *e-PCR* (16), a tool developed at NCBI and installed locally. *e-PCR* is performed with the following options:-d50-500 (*-d*=‘default size range’), -n2 (*-n=*‘Max mismatches allowed per primer’), -g2 (*-g=*‘Max number of indels allowed per primer’), -t3 (*-t*=‘output in tabular format’). Amplicons are selected and further processed in the following steps only if the total number of mismatches and gaps is less or equal to 2.
2. Retrieval of the matching sequences, annotation of their record ID and extraction of the relative sub-regions (amplicons) obtained by *e-PCR*;
3. If a probe is present, alignment of the probe and amplicon sequences is performed by *blast2seq*, a sequence comparison tool of the NCBI-BLAST package (17);
4. Species assignment of the amplicon sequences by querying the Taxonomy database (18, 19) with the target record ID;
5. Generation of the output as TAB delimited file with all metadata to populate/update the database.

### Web interface construction and features

The JRC GMO-Amplicons interface is a Ruby on Rails application (http://rubyonrails.org/) integrated within the EU-RL GMFF public website (http://gmo-crl.jrc.ec.europa.eu/). It is backed by a local PostgreSQL database which is automatically updated by local scripts that parse the TAB delimited files generated in the annotation pipeline above.

The application also integrates, as a frame, a local copy of the SequenceServer software (Priyam A, Woodcroft BJ, Rai V & Wurm Y SequenceServer: BLAST searching made easy, in preparation, http://sequenceserver.com) to add the BLAST-based similarity search functionality. Modifications to the original source code (version 0.8.7) were limited to visual elements, color scheme and security improvements. BLAST datasets are generated using the *makeblastdb* command of the NCBI-BLAST package (17).

### Clustering

Clustering of amplicon sequences in order to generate the non-redundant amplicon set used for BLAST is accomplished by running *cd-hit*, version V4.6.1 (20) with the following options: -aL = 0.9 (alignment coverage for the longer sequence), -aS = 0.9 (alignment coverage for the shorter sequence).

## Results and discussion

### Analysis of pipeline output

Using the reference methods included in the GMOMETHODS database and the sequences from the selected public resources, the pipeline produced a set of more than 240 000 amplicons, which ultimately populate the JRC GMO-Amplicons database. Out of the available 140 methods in the GMOMETHODS database, 132 (94%) were able to pick up at least one sequence in the sequence datasets and 128 (91%) had a perfect match in the annealing region of the primers with their corresponding DNA target. The set of detected amplicons sequences can be clustered in 722 distinct groups each composed of 100% identical sequences (184 groups by setting both 90% identity and 90% sequence length coverage for each sequence pair comparison). This set of 722 sequences defines a ‘non-redundant’ set of amplicons that can be queried by similarity search tools.

**Figure 1.**
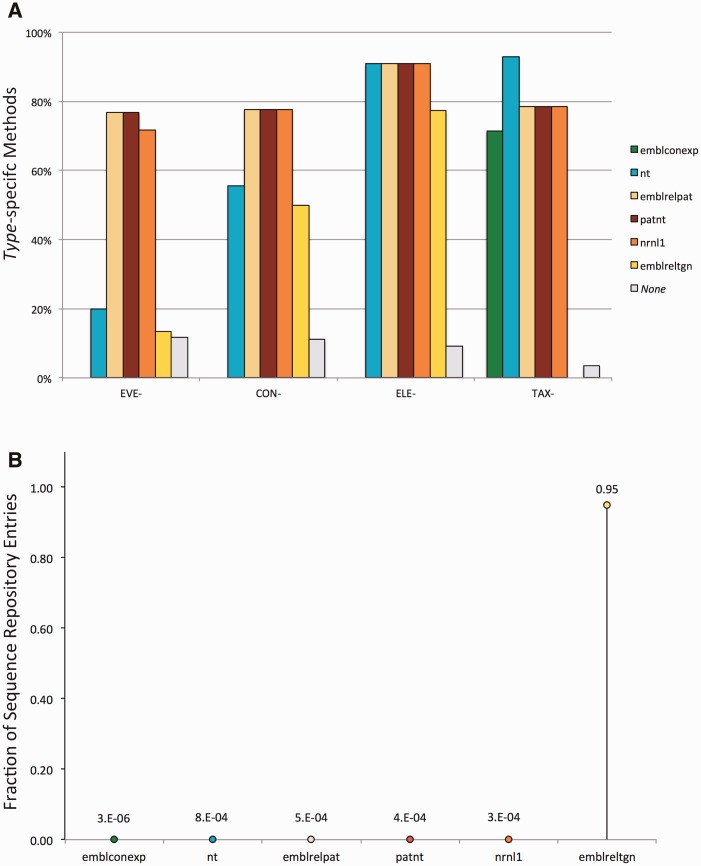
Evaluation of the consistency of the pipeline output. Detected amplicons are evaluated with respect to the dataset of origin and the type of methods used to detect them, revealing a non-random distribution of the amplicons consistent with both the method type and the dataset composition. (**A**) Percentage of methods from the GMOMETHODS database, grouped by specificity, that generate *in silico* at least one amplicon in the different sequence datasets; (**B**) Fraction of records in each of the analysed datasets that can be detected by the methods from the GMOMETHODS database. Only amplicons with 0 gaps and 0 mismatches in primers and probe annealing were taken into account.

Element-specific methods are expected to target many sequences, including those of the elements' donor organisms, in all the input datasets. In contrast, construct- and (to a larger extent) event-specific methods are expected to target fewer sequences, which should be found mostly in the patent-related datasets. In order to verify the consistency of the matches between the datasets and methods we used, amplicons were analysed with respect to the dataset of origin and the type of methods used to detect them. These analyses revealed a non-random distribution of the amplicons consistent with both the method type and the nature of the datasets, in agreement with our hypothesis, as highlighted in Figure 1.

Figure 1A shows the percentage of methods, classified by specificity, that generate *in silico* at least one amplicon in the different sequence datasets. It can be observed that:

- GM-specific methods (i.e. event-, construct-, element-specific) do not detect any sequence in the ENA plants contigs dataset (*emblconexp* bars in Figure 1A), where GM-sequences are not expected to be present.
- Conversely, taxon-specific methods do not detect any sequence in the ENA transgenic dataset (*emblreltgn* bars in Figure 1A), where plant genomic sequences are presumably not occurring.
- Most of the event-specific methods find putative amplicons in the patent-related sets (i.e. *patnt*, *emblrelpat*, *nrnl1* bars in Figure 1A) and only 20% in the NCBI nt section, thus reflecting the fact that GMO sequences are usually patented and not deposited in the publicly available DNA sequence resources.
- All the element-specific methods, that are not specific for individual GMOs, detect sequences in all the analysed datasets except the ENA plants contigs (*emblconexp* bar in Figure 1A).
- The distribution for each type of GM-specific method (i.e. event-, construct-, element-specific) is practically identical in the three patent-related datasets (i.e. *patnt*, *emblrelpat*, *nrnl1*), with the exception of three recently added event-specific detection methods (QT-EVE-GM-012, QT-EVE-GM-013 and QT-EVE-GM-001), whose targets were not present at the time of the last *nrnl1* release (2013).

Figure 1B, conversely, shows the fraction of records in each of the analysed datasets that can be detected by the methods from the GMOMETHODS database. Consistent with the nature of these datasets, it can be shown that:

- 95% (19 812 out of 20 800) of the *emblreltgn* records are detected by at least one of the GM-specific methods. This suggests that the methods available in the GMOMETHODS database cover 95% of the public nucleic acid sequences classified as transgenic. This fraction is considerably higher than in the other datasets, where only an insignificant portion (on average about 1/10 000) were detected.
- Plant contigs records (*emblconexp* dataset) are not expected to be related to GMOs. In fact, only few sequences (1/1 000 000) are positive to detection methods and all of these are, as expected, taxon-specific methods.

**Figure 2.**
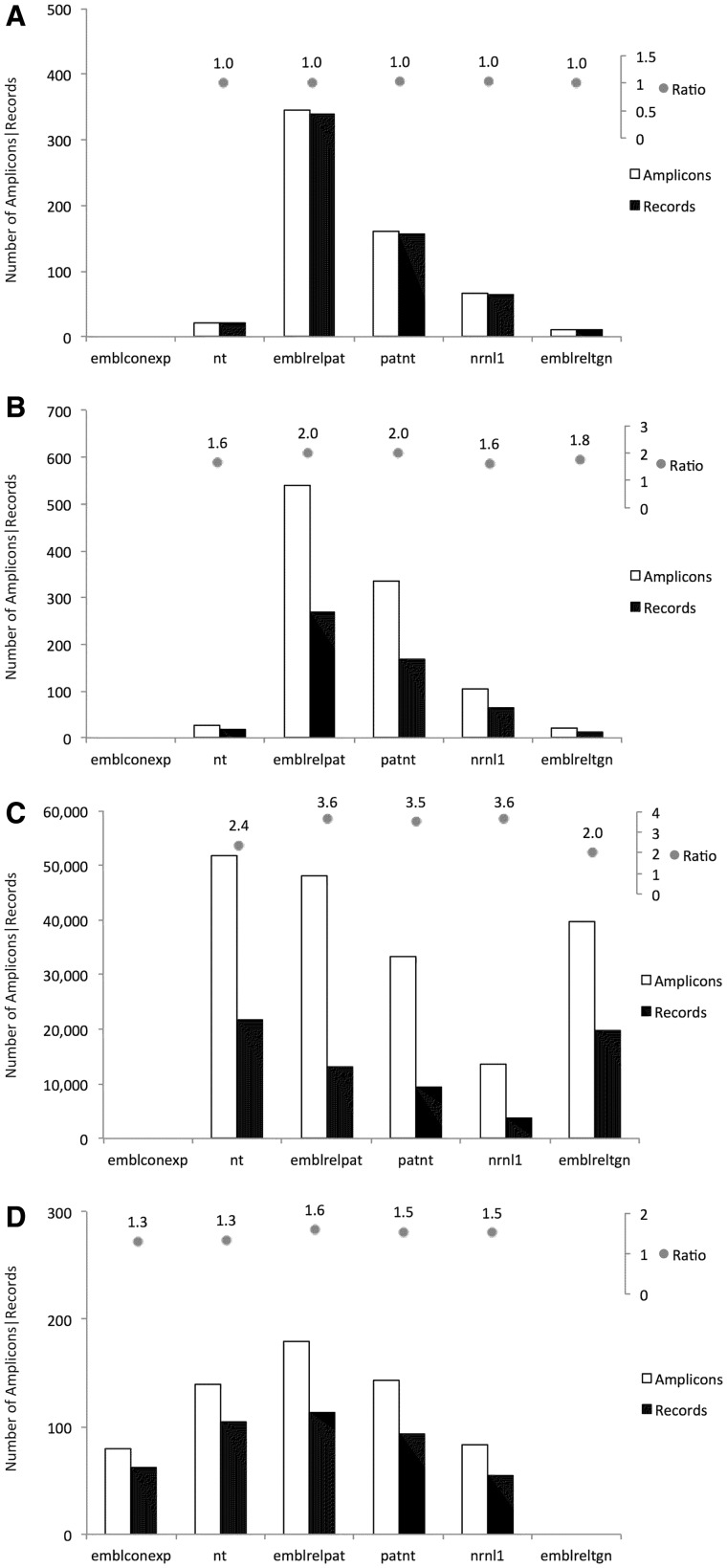
Number of amplicons and number of target records found in the selected datasets. For each type of detection methods, amplicons and target records are plotted as bars: (**A**) Event-specific methods; (**B**) Construct-specific methods; (**C**) Element-specific methods; (**D**) Taxon-specific methods. On top of each panel, the ratio of these numbers is also reported. Only amplicons with 0 gaps and 0 mismatches in primers and probe annealing were taken into account.

Some records were found to produce an amplicon with more than one method. Once again, this trend differs according to the sequence dataset and the specificity of the method. As shown in Figure 2 event-specific (A), element-specific (B), construct-specific (C) and taxon-specific (D) methods display distinctive patterns of PCR amplification predictions in the different datasets:

- Event-specific methods produced a single amplicons for each retrieved record (Figure 2A), thus in a 1:1 relationship, as they target regions that are unique in each record. The only exceptions relate to different methods targeting the same GM event, as for example in record nrnl1:NRN_AX685147 which was retrieved by the QL-EVE-BN-002 and QT-EVE-BN-004 methods, both specific for the detection of oilseed rape event GT73.
- Element-specific methods produced *in silico* hundreds of thousands of amplicons (about 1,000 times more than the other ones, see Figure 2B) as they were designed to detect sequences of elements commonly used in GMOs development. Moreover, a single record can be targeted by different element-specific methods if its recombinant molecule contains more than one element. As expected, element-specific methods target a lot of sequences that correspond to ‘natural elements’, i.e. elements existing in the sequence of their donor organism outside of their use in GMOs. For example, more than 40 000 amplicons were found for the two detection methods against the neomycin phosphotransferase II (*nptII*) gene, identifying both the natural counterpart and a panel of cloning vectors.
- Construct-specific methods (Figure 2C) share behaviour similar to the element-specific methods, but since they target the junction between elements in synthetic constructs, they generate a smaller number of amplicons. Moreover, most of the records are retrieved from patent-related datasets, as synthetic constructs are often patented.
- Taxon-specific methods (Figure 2D) may, in some instances, target the same record, since different regions of the same gene have sometimes been used for the development of multiple methods. One example is the soybean lectin gene (*Le1*), where distinct regions are targeted by different soybean-specific methods.

All the above observations showing the non-random but logical selection of records indicate a successful generation of a consistent, enriched, GMO-related set of sequences. Moreover, according to these findings, the number of false positives and negatives is expected to be very low.

### Database access

**Figure 3.**
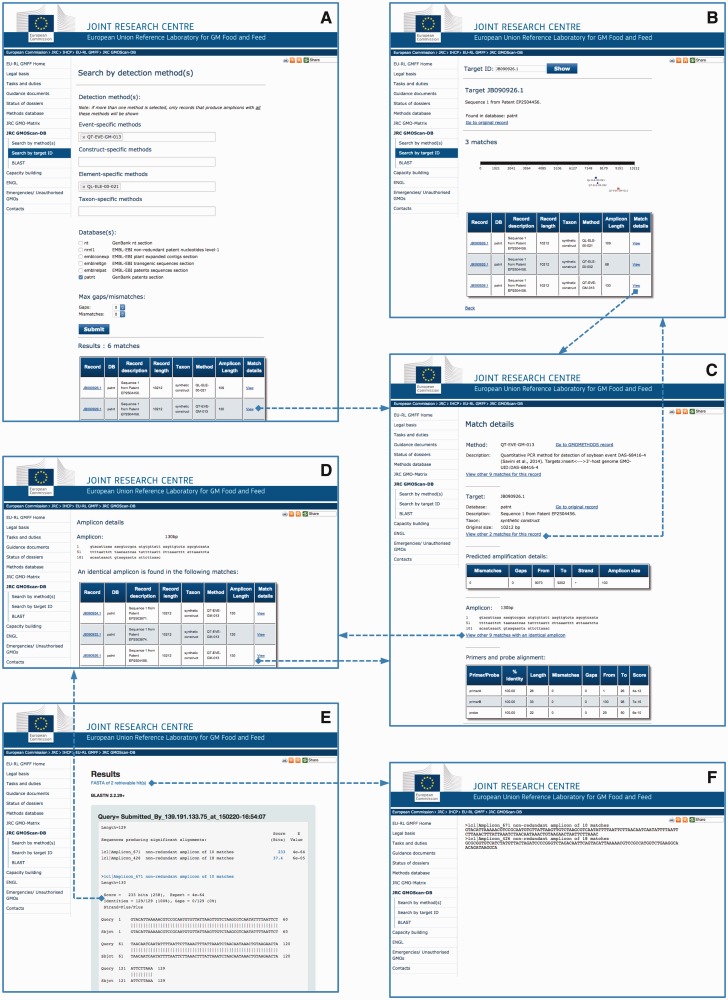
JRC GMO-Amplicons web interface overview. Screenshots of the JRC GMO-Amplicons web interface, showing the different entry points and integrated views to data, which can be easily explored by browsing through web links. See text for details.

The JRC GMO-Amplicons is accessible through a web portal (Figure 3), where queries can be performed according to the methods (Figure 3A) or the identification number of the target record (Figure 3B). The system provides a list of matches, and in the latter case a graphical representation of the amplicons mapped on the target. From the results page it is possible to inspect the match details (Figure 3C) or further examine the ‘amplicon detail page’ (Figure 3D), i.e. a page reporting a list of identical amplicons retrieved from all the records of the scanned sequence resources.

The non-redundant amplicons set and the amplicon sets derived from the different individual datasets can be searched by BLAST (Figure 3E). Matching records can be downloaded for further analyses (Figure 3F).

### Examples of JRC GMO-Amplicons use cases

#### Information mining for poorly characterized GMOs

GMO-related sequences are expected to be present in public databases, but are hard to retrieve, in particular from patent-related datasets. The JRC GMO-Amplicons database facilitates the identification of the full GMO sequences and of the corresponding amplicons in the patent datasets and, therefore, supports control laboratories in the detection and identification of unauthorized GM events. The database can also aid annotating poorly described GM events or recognizing new GM-related sequences in public repositories. Some examples of applications for the JRC GMO-Amplicons database in food/feed control are provided below.

##### Characterization of GM rice event LLRICE62

**Figure 4.**
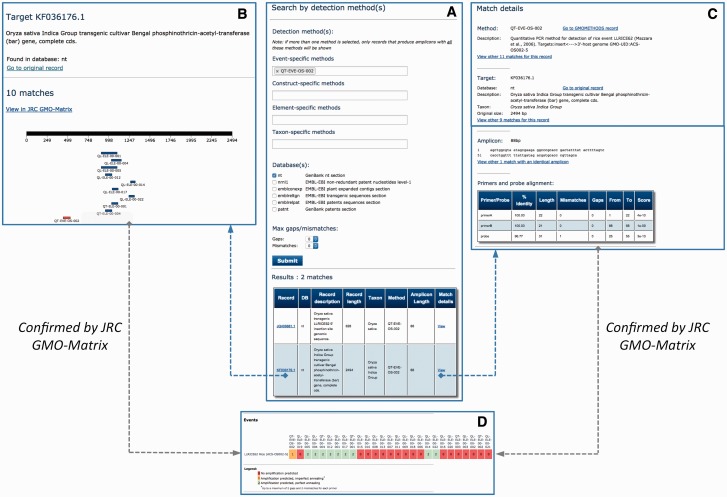
Retrieval of amplicons and target records related to GMO events. Integration of views showing the use of JRC GMO-Amplicons to retrieve GM rice event LLRICE62 targets and corresponding amplicons. Results are in agreement with those obtained by the JRC GMO-Matrix application. See text for details.

GM rice event LLRICE62 (Unique identifier ACS-OS002-5) is an *Oryza sativa* transgenic insect-resistant line that is not authorized in the EU market. A quantitative PCR method for LLRICE62 detection has been developed and included in GMOMETHODS (QT-EVE-OS-002, 21). The 5' flanking region of the LLRICE62 event has been published in 2012 (GenBank: JQ406881.1, 22) while the sequence of the full event generated by NGS technology has been made publicly available in 2013 (GenBank: KF036176.1, 23). These sequences can be easily identified by querying the JRC GMO-Amplicons database with the QT-EVE-OS-002 method (Figure 4A). Moreover, by browsing the match detail panel of KF036176.1, it is possible to identify other element-specific methods (Figure 4B) targeting the event, as confirmed by the JRC GMO-Matrix (Figure 4D). The amplicon produced by the QT-EVE-OS-002 method (Figure 4C) is annotated in the database with one mismatch in the probe, as also pointed out by the JRC GMO-Matrix (Figure 4D). Finally, other sequences in the patent databases can be identified in the query as producing the same amplicon: all of them are part of patents claiming the development of glufosinate tolerant rice strains, of which LLRICE62 is a member (see for example AR2338850.1 of the *patnt* database, found in the granted patent US-6468747-B1). Together, these analyses show the use of the JRC GMO-Amplicons database for easily identifying new relevant sequences in public repositories.

##### Characterization of GM rice event TT51-1

GM rice event TT51-1 (also known as rice Bt63) is an *Oryza sativa* transgenic insect-resistant line that is not authorized in the EU market. Only three qualitative methods of the GMOMETHODS database (QL-CON-00-009, QL-CON-00-007 and QL-ELE-00-016) are described for detecting this event (24–26). In the JRC GMO-Amplicons interface four amplicons from sequence EU880444.1 (*transgenic* section of the ENA database) are identified when using these methods in a simultaneous query. The target record shows two putative amplicons for each of the selected methods and, in addition, several other ones from element-specific methods, all of them confirmed by the JRC GMO-Matrix decision support tool. These findings support the hypothesis of the presence of multiple copies of the Bt63 target determined in the development of the selected construct-specific methods (27). Sequence EU880444.1 was published as GM rice event TT51-1 in 2010 (28). Yang *et al.* (29) reported a new version that is slightly different. The pipeline did not find this newer sequence because it was made available only as supplementary material to the article and never submitted to public databases.

#### Analysis of unexpected detection methods results

**Figure 5.**
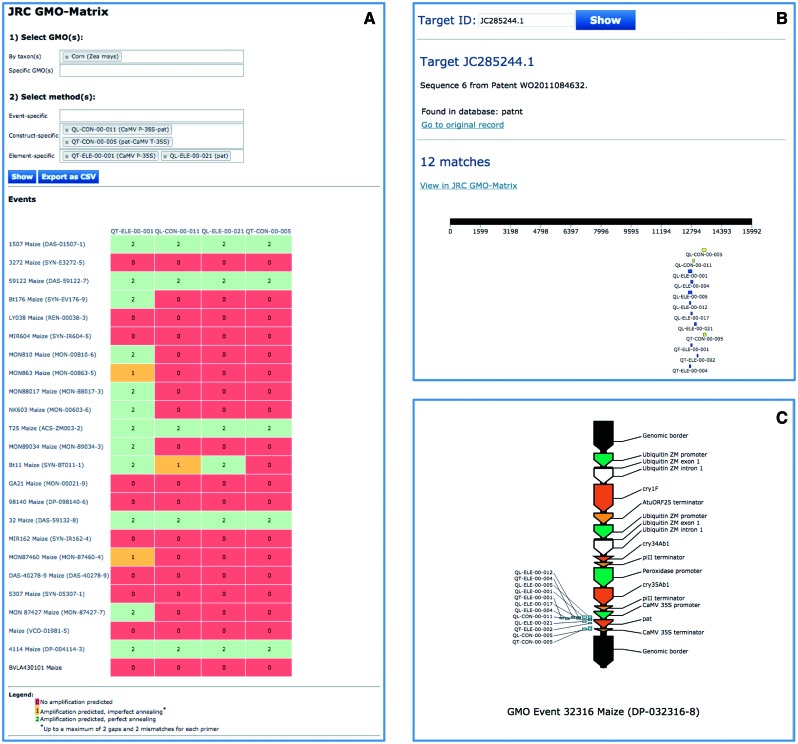
Use of JRC GMO-Amplicons to assist interpretation of screening results. A case-study of detection and identification of unauthorized GM maize event in food/feed products. A maize sample was found positive to specific methods QL-CON-00-011 (junction between the Cauliflower Mosaic Virus 35S promoter and the *S. viridochromogenes pat* gene), QT-CON-00-005 (junction region between the *pat* gene from and the Cauliflower Mosaic Virus 35S terminator) and to two element detection methods, QT-ELE-00-001 (Cauliflower Mosaic Virus 35S promoter) and QL-ELE-00-021 (*pat* gene). The JRC GMO-Matrix (**A**) predicts 5 GM maize events as potential events positive to all of them, but the sample is negative for the corresponding event-specific methods. By querying the database with these methods and focusing on the unique records of the *patnt*, sequence, JC285244.1 from patent WO2011084632 (33) is found (**B**). According to the annotation, this sequence corresponds to the ‘*complete sequence of the insert and flanking regions of event DP-032316-8*’, a good candidate for which the sample can be easily tested, as the patent describes also a detection method for this event. JC285244.1 was fully characterized (**C**), confirming that it is the full sequence of DP-032316-8 maize event.

Using the JRC GMO-Amplicons, it is possible to identify GM-related sequences that are positive for the elements detected in the analysed samples but negative for the event-specific methods included into the GMOMETHODS database. A case-study example is shown in Figure 5, where a maize sample was found positive for two construct specific methods, QL-CON-00-011 (30) and QT-CON-00-005 (29) and to two element detection methods, QT-ELE-00-001 (31) and QL-ELE-00-021 (32). Through the JRC GMO-Amplicons it was possible to identify a new patent sequence record (33) and to download it for further characterization.

#### Sequence similarity searches

The JRC GMO-Amplicons gives the possibility to search for similarity by NCBI-BLAST (17). As previously described (see Figure 3E and F), the non-redundant amplicons set and the amplicon sets derived from the different individual datasets can be searched by using a user-defined sequence as query. Through the interface, it is possible to adjust default algorithm parameters, like the e-value (i.e. the expectation value threshold for saving hits) or the penalties for mismatches and introduced gaps. It is also possible to choose to run the application in BLASTN or TBLASTX mode, i.e. by searching nucleotide databases using a nucleotide query or by searching translated nucleotide databases using a translated nucleotide query, respectively. The output is in the format of the classical NCBI BLAST output, with the additional option of downloading all matching amplicons for further analyses. Overall, this functionality is very useful in cases when control laboratories have obtained a sequence from an ‘unknown sample’ by direct sequencing of a PCR amplicon or by DNA walking anchored on GM elements, the latter already implemented in the context of identification of unauthorized GMOs (34).

## Conclusion and future direction

To our knowledge, the JRC GMO-Amplicons is the first attempt at making sequences specifically related to GMOs publicly available. The JRC GMO-Amplicons web application is a gateway to mine the results of a pipeline that regularly screens public nucleotide sequence databanks, including patents and available whole plant genomes, through *in silico* determination of PCR amplification with the reference methods of the GMOMETHODS database.

A panel of five different public nucleic acid sequence data sets were selected for screening, even if potentially redundant, to have the most comprehensive set of possible target records and corresponding amplicons. According to our findings, this objective was achieved: 95% of the sequences classified as transgenic in the public databases were picked up by at least one method, and 91% of the selected methods had at least one target record in the selected databases. In addition, target records and corresponding amplicons were distributed in a non-random manner that was consistent with both the method type and the dataset of origin. This strongly suggests that the procedure of pooling a consistent, enriched, GMO-related set of sequences has been successful and specific.

The modular infrastructure behind the implementation of the JRC GMO-Amplicons allows to efficiently maintain up-to-date information when new detection methods and data resources become available. The platform could also allow the straightforward implementation of different tools for scanning patent resources or adding new annotations to the identified sequences. For example, it is scheduled to annotate amplicons extracted from patented sequence records with the original patent publication numbers. Moreover, a similarity clustering procedure will be implemented on the original target records in order to create a non-redundant dataset of GMO-related targets. Finally, work is under way to allow *in silico* determination of PCR amplification with custom primers.

The JRC GMO-Amplicons, together with the JRC GMO-Matrix, will help bridging the gap between the DNA-based detection methods and the important information contained in the GMO DNA sequences themselves, often difficult to obtain.

*Conflict of interest*. None declared.
